# Severe Acute Respiratory Syndrome Coronavirus 2 and Male Reproduction: Relationship, Explanations, and Clinical Remedies

**DOI:** 10.3389/fphys.2021.651408

**Published:** 2021-04-14

**Authors:** Jia Xu, Liting He, Yuan Zhang, Zhiyong Hu, Yufang Su, Yiwei Fang, Meilin Peng, Zunpan Fan, Chunyan Liu, Kai Zhao, Huiping Zhang

**Affiliations:** Institute of Reproductive Health, Center for Reproductive Medicine, Tongji Medical College, Huazhong University of Science and Technology, Wuhan, China

**Keywords:** SARS-CoV-2, COVID-19, male reproduction, immunology, viral orchitis

## Abstract

Coronavirus disease 2019 (COVID-2019) caused by severe acute respiratory syndrome coronavirus 2 (SARS-CoV-2) has been an ongoing pandemic and worldwide public health emergency, having drawn a lot of attention around the world. The pathogenesis of COVID-19 is characterized by infecting angiotensin-converting enzyme 2 (ACE2)-expressing cells, including testis-specific cells, namely, Leydig, Sertoli, and spermatogenic cells, which are closely related to male reproduction. This leads to aberrant hyperactivation of the immune system generating damage to the infected organs. An impairment in testicular function through uncontrolled immune responses alerts more attention to male infertility. Meanwhile, the recent clinical data indicate that the infection of the human testis with SARS-CoV-2 may impair male germ cell development, leading to germ cell loss and higher immune cell infiltration. In this review, we investigated the evidence of male reproductive dysfunction associated with the infection with SARS-CoV-2 and its possible immunological explanations and clinical remedies.

## Introduction

Coronaviruses, which are common zoonotic human pathogens, are at the forefront of global news again after the incidences of the severe acute respiratory syndrome (SARS) during 2003 (Cinatl et al., 2003) and the Middle East respiratory syndrome (MERS) during 2012 (Zumla et al., 2015). Now, a newly discovered coronavirus, called SARS coronavirus 2 (SARS-CoV-2) (Wu et al., 2020), a member of the Coronaviruses family, was shown to swiftly become pandemic resulting in a rapidly growing number of patients in many countries and territories, including the United States, Canada, Japan, Singapore, Australia, France, Spain, Germany, the United Kingdom, India, Iran, Italy, and Africa. This situation posed a serious public health issue, taking the World Health Organization (WHO) by surprise; therefore, the WHO classified the coronavirus disease 2019 (COVID-2019) as a pandemic. However, efforts to identify effective therapeutic treatments have been hampered by our limited understanding of the host immune response to this fatal disease. To date (January 8, 2021), there have been 37 million confirmed cases and 1 million deaths reported globally (World Health Organization, 2020a). Clinical data from China and elsewhere indicated that the common manifestations of COVID-19 are fever, dry cough, and fatigue (Wang D. et al., 2020), with 80% of patients presenting mild-to-moderate symptoms or appearing asymptomatic, whereas 15–20% of patients exhibit severe symptomology (Huang et al., 2020a; Xu et al., 2020; Young et al., 2020). Consequently, the real figure of undetected individuals with mild symptoms tends to be higher than the official data, a fact that could lead to uncontrolled transmission.

Briefly, SARS-CoV-2 is part of the Coronaviruses family, belonging to the *Betacoronavirus* genus, typically infecting only mammals (Zumla et al., 2015). Other human pathogenic coronaviruses, including SARS coronaviruses (SARS-CoVs) and MERS coronaviruses (MERS-CoVs), are also betacoronaviruses. The recently released sequence of SARS-CoV-2 indicated that SARS-CoV-2 is highly homologous to SARS-CoV, sharing about 79% of their genome (Wan et al., 2020; Zhou et al., 2020). The similarities shared between SARS-CoV and SARS-CoV-2 were demonstrated to be mostly in the immunogenic component of coronaviruses, involved in the binding to the same angiotensin-converting enzyme 2 (ACE2) receptors. These receptors are well-known entry receptors for SARS-CoV, suggesting that SARS-CoV could infect ACE2-expressing cells (Hoffmann et al., 2020; Zhou et al., 2020). Interestingly, previous investigations reported that ACE2 was expressed in Leydig cells of rat testis and in Leydig cells and Sertoli cells (SCs) of the human testis (Douglas et al., 2004; Xu et al., 2006). Likewise, recent bioinformatics analysis revealed that ACE2 was highly expressed not only on alveolar epithelial cells (Zhao et al., 2020) but also on the Leydig cells in the rat testis and on Leydig cells and SCs in the human testis (Douglas et al., 2004; Watermeyer et al., 2006; Anguiano et al., 2017). It is worth noting that single-cell RNA-sequencing data of adult human testis indicates that the mRNA expression of ACE2 was expressed in both germ cells and somatic cells and that the positive rate of ACE2 in the testes of infertile men was higher than normal, which cautions that SARS-CoV-2 may cause reproductive disorders through a pathway activated by ACE2 and that men with reproductive disorder may easily be infected with SARS-CoV-2 (Shen et al., 2020). On the other hand, testicular Leydig cells and SCs are known to be closely related to testicular function and male reproduction. Of note, viruses such as SARS-CoV, mumps virus (MuV), and Zika virus (ZIKV) have been reported to affect Leydig cells, destroying the blood–testis barrier (BTB) and breaking the seminiferous epithelium through the formation of viral orchitis (Xu et al., 2006; Govero et al., 2016; Ma et al., 2016; Wu et al., 2016). Therefore, we hypothesized that infection with SARS-CoV-2 might affect testicular function and result in the dysfunction of the male reproductive system.

## Immune Privilege in the Testis

**FIGURE 1:**
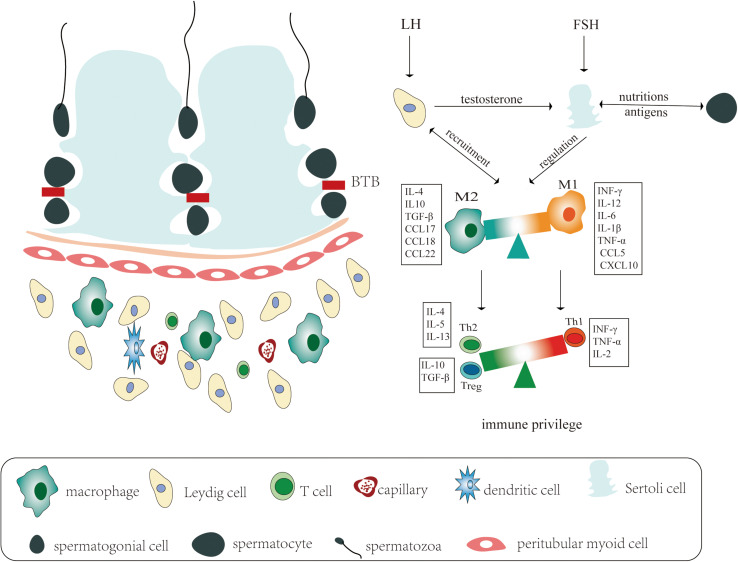
Schematic representation of the immune privilege in the testicular immune microenvironment. **Left:** The adult mammalian testis consists of two distinct structurally regions: the seminiferous tubules and the interstitial spaces between tubules, corresponding to the function of spermatogenesis and steroidogenesis, respectively. The seminiferous tubes consist of various developing germ cells surrounded by Sertoli cells, while the testicular interstitial spaces are composed of most types of immune cells and testis-specific Leydig cells. **Right:** Under physiological conditions, the mammalian testicular immune privilege is correlated with blood–testis barrier (BTB), immune cells in interstitial spaces, and endocrine regulation of testicular function. The immunological milieu of the testis is regulated by luteinizing hormone (LH), which acts on the Leydig cells, and follicle stimulating hormone (FSH), which acts on the Sertoli cells. In response to stimulation by LH, the Leydig cells produce testosterone, which are necessary to support mature Sertoli cell functions. The Leydig cells are responsible for recruiting macrophages into the interstitial tissue. Macrophages are functionally divided into two disparate populations: pro-inflammatory function (M1) and anti-inflammatory actions (M2). The M2 populations have an immunoregulatory role in maintaining immune privilege by producing cytokines and chemokines including IL-4, IL-10, TGF-β, CCL17, CCL18, and CCL22, whereas during inflammation, M1 drastically recruits pro-inflammatory immune cells by releasing cytokines or chemokines including IFN-γ, IL-6, TNF-α, IL-1β, CCL5, and CXCL10. The Sertoli cells and macrophages regulate the development of lymphocytes circulating through the testis, promoting the activity of regulatory T cells (e.g., Th2 cells and T-reg cells) and suppressing the activity of Th1 cells.

The adult mammalian testis consists of two structurally distinct regions: the seminiferous tubules and the interstitial spaces between tubules (Figure 1), corresponding to the function of spermatogenesis and steroidogenesis, respectively. Under physiological conditions, the mammalian testis presents a unique immunological milieu, an immune privilege, where germ cells are protected from endogenic immune attack, including the immune response from not only allo-antigens but also auto-antigens (Hedger, 2015; Monika et al., 2017). The testicular immune privilege has been correlated with the coordination of systemic immune tolerance, active local immunosuppression, and the local anatomy of the testis. Of course, testicular defense mechanisms have been also shown to contain the counteraction of invading ectogenic microbial pathogens derived from circulating blood or ascended from the genitourinary tract when the testicular immune privilege has to be overcome. However, an excessive immune response from accumulation of systemic immune cells or perturbing local immune homeostasis between the testicular immune tolerance and effect of the testicular immune response could lead to inflammatory-based male infertility (Mellor and Munn, 2006; Hedger, 2015; Fijak et al., 2018), just as HIV and MuV infect human testicle and cause testicular damage leading to male infertility (Philip et al., 2006; Garolla et al., 2013). Herein, we highlight some of the mechanisms that are correlated with the establishment, maintenance, and disruption of the testicular immune privilege.

### Sertoli Cells and Blood–Testis Barrier

SCs make up the structural platform of seminiferous tubules with all stages of spermatogenesis inside. They could also maintain the testicular immune privilege by skewing immune responses through producing immunosuppressive molecules (Fallarino et al., 2009; Zhao et al., 2014) and immunosuppressive cytokines, such as transforming growth factor β (TGF-β), tumor necrosis factor α (TNF-α), and activins (De et al., 1993; Avallet et al., 1994; Meehan et al., 2000), found in systemic and local testicular inflammation. The BTB, which is formed by various junctions, including tight junctions connecting adjacent SCs and gap junctions, separates germ cells from the immune cells present in interstitial spaces, protecting against immune cell recognition of these germ cells.

### Interstitial Cells and Immune Privilege in the Testis

The human testis is composed of a variety of cell types including most types of immune cells—like testicular macrophages (TMφ), dendritic cells (DCs), and lymphocytes—and testis-specific Leydig cells. TMφ are a predominant population of cells in the interstitial space of testis, representing approximately 20% of all testicular interstitial cells in the normal testis (Hedger, 2002). Under physiological conditions, TMφ have been reported to constitutively secrete low levels of pro-inflammatory cytokines [TNF-α and interleukin-12 (IL-12)] in response to inflammatory stimuli, whereas maintenance of the immune privilege requires the concomitant secretion of high amounts of anti-inflammatory cytokines, such as IL-10, as shown in previous studies (Gerdprasert et al., 2002; Bhushan et al., 2015). In a special condition, TMφ have dynamic properties and can develop into either pro-inflammatory function (M1) or anti-inflammatory actions (M2). During a viral infection, exogenous signals, such as cytokine signaling or pathogen recognition, have been shown to propel macrophage polarization from the M2 to M1 phenotype. Resident M2 populations are known to have an immunoregulatory role in maintaining the immune privilege and trophic functions, particularly in Leydig cells, by producing cytokines and chemokines including IL-4, IL-10, TGF-β, C-C motif chemokine ligand 17 (CCL17), CCL18, and CCL22. In contrast, during acute and chronic inflammation, M1 inflammatory subsets have been reported to drastically recruit immune cells to the site of infection, skewing the cytokine balance and stimulating an inflammatory response by releasing cytokines or chemokines including interferon γ (IFN-γ), IL-6, TNF-α, IL-1β, CCL5, and C-X-C motif chemokine ligand 10 (CXCL10) (Gerdprasert et al., 2002; Mantovani et al., 2004; Biswas and Mantovani, 2010; Geissmann et al., 2010; Locati et al., 2013; Murray et al., 2014; Li et al., 2019).

DCs, being a tenth of the quantity of macrophages, are usually known as phagocytes particularly involved in antigen presentation and in causing adaptive immunity, Moreover, DCs could not only assist T-cells resistant to antigens and minimize autoimmune responses but also activate antigen-specific T-cells, thus initiating a series of autoimmune responses in the testis leading to immunological infertility (Rival et al., 2006; Jacobo et al., 2009).

Lymphocytes represent 10–20% of all leukocytes cells, with the majority of testicular lymphocytes being T-cells composed mainly of CD8 +, CD4 +, and regulatory T (T-reg) cells (Hedger and Meinhardt, 2000; De Rose et al., 2013). Significantly increased numbers of T-cells are the hallmark of the perturbed immunological balance during testicular inflammation. Skewing of the testicular T-cell subsets to T-reg cells in autoimmune-related orchitis (Jacobo et al., 2009, 2011) indicates that testicular T-reg cells are powerful immunosuppressive cells, promoting peripheral tolerance and adjusting the tolerogenic versus the autoimmune response to sperm antigens. Besides, the count of lymphocytes within the testicular interstitial space has been tightly related to the number and activity of the population of macrophages by releasing cytokines (Hedger and Meinhardt, 2000).

Leydig cells are another large proportion of interstitial cells, generally in close apposition to immune cells. The main function of Leydig cells is to synthesize testosterone for both spermatogenesis and extratesticular androgen target organs. In addition, Leydig cells can be stimulated to produce immunoregulatory cytokine, which in turn recruits immune cells, most likely macrophages, resulting in the disruption of the immune homeostasis in testis, which affects spermatogenic cell development (Fijak et al., 2011; Loveland et al., 2017).

### Endocrine Regulation of Testicular Function and Immune Privilege

The testosterone synthesized by Leydig cells has been reported to suppress both the systemic and testicular immune responses to auto-antigens (Cutolo, 2009; Fijak et al., 2011; Avellar et al., 2019). Moreover, testosterone could act on SCs through the androgen receptor (AR) expressed on these cells. Mouse models of cell-specific AR knockout in SCs were demonstrated to lead to the impairment of the testicular immune privilege, leading to spermatogenesis arrest (Page et al., 2006). On the other hand, the presence of inflammatory factors in the testis can also in turn affect the function of Leydig cells, thus affecting testosterone secretion, when orchitis occurs. Another hormone, the follicle-stimulating hormone (FSH) produced by the pituitary gland, has also been reported to regulate the proliferation of SCs (Barakat et al., 2008; Cannarella et al., 2019; Meroni et al., 2019), which in turn induce macrophage invasion into the seminiferous tubules by secreting an array of cytokines, leading to spermatogonia apoptosis (Wang et al., 2013).

## The Immune Responses Associated With Severe Acute Respiratory Syndrome Coronavirus 2 in Testis

A few recently published studies investigated the immune response or cytokine-relative clinical manifestations of patients infected with COVID-19. In general, infection with SARS-CoV-2 could initiate an immune response, resulting in many kinds of immune cytokine be released. When an uncontrolled immune response, even cytokine storm and immunodepletion, was observed, the patient will present with more severe symptoms and might have detrimental tissue damage (Cao, 2020).

As we know, the testis is a special immune organ maintained by its dependence on the immune cells in the testicular stroma. As such, lymphopenia and immunodepletion caused by SARS-CoV-2 infection could impair the number of immune cells recruited from the systemic to testicular tissue, disturbing the balance between the immune privilege and immunosuppression that exists in the testis. Besides, a cytokine storm, pro-inflammatory cytokines, and chemokines from the systemic tissue could also trigger immune responses in the testis, destroying the testicular tissue and increasing its susceptibility to pathogens. One unfortunate conclusion of all the above would be that the impairment of the testicular function induced by an infection with SARS-CoV-2 cannot be ignored from an immunological point of view.

## Clinical Evidence of Potential Male Reproduction Dysfunction of Patients Infected With Severe Acute Respiratory Syndrome Coronavirus 2

The testis, with its immune tolerance mechanism maintained by both local immunosuppression and systemic immune responses, is a special immune organ that is susceptible to many microbial infections that might damage its precision system, causing immune-mediated damage (Rival et al., 2006; Liu et al., 2018; Stassen et al., 2018). Various viruses, such as MuV, HIV, and ZIKV, are known to have tropism for the male reproductive system, particularly the testis; therefore, infection with any of them might induce damage to the testis, resulting in male infertility (Liu et al., 2018). To date, there have been some clinical data demonstrating the relationship between SARS-CoV-2 and the human testis (Table 1); however, the outcomes reported have been paradoxical.

**TABLE 1:** Viral, biological, and clinical evidences showing thecorrelation between SARS-CoV-2 and potentialmale infertility.

Research content	Results	References
SARS-CoV-2 sequence	Highly homologous with SARS-CoV; have similarities with ACE2	Hoffmann et al., 2020; Ma et al., 2020; Wan et al., 2020; Zhou et al., 2020
ACE2 expression in testis	Positive in Leydig cells, Sertoli cells, and germ cells	Douglas et al., 2004; Watermeyer et al., 2006; Anguiano et al., 2017; Ma et al., 2020; Shen et al., 2020;
scRNA-seq profiling of the human testis	ACE2 is enriched in spermatogonia, and Leydig and Sertoli cells	Shen et al., 2020; Wang and Xu, 2020
Sex hormones changes	LH increase; T/LH decrease	Ma et al., 2021
SARS-CoV-2 RNA in serum	Positive (1/5, 1/12, 39/95)	Lescure et al., 2020; Young et al., 2020; Zheng et al., 2020
SARS-CoV-2 RNA in urine	Positive (4/58, 3/48, 1/67)	Cao, 2020; Ling et al., 2020; Wang L. et al., 2020; Zheng et al., 2020
SARS-CoV-2 RNA in semen	Positive (6/38)	Li D. et al., 2020
SARS-CoV-2 RNA in testis	Positive (2/5, 0/12)	Ma et al., 2020; Song et al., 2020
Impaired spermatogenesis	Germ cell loss in testicular section; RNA-seq data shows down-regulated gene enriched in spermatogenic cells and reproduction	Ma et al., 2020
Immunological manifestations in testis	Higher T-cells and macrophages in testis; increased levels of IL-6, IL-8, TNF-α, and MCP-1 in semen	Li D. et al., 2020; Ma et al., 2020; Gacci et al., 2021

*SARS-CoV-2, severe acute respiratory syndrome coronavirus 2; ACE2, angiotensin-converting enzyme 2; LH, luteinizing hormone; T, testosterone.*

To overcome this, we would address some lessons learned from other viral diseases that could infect the testis and lead to viral orchitis or male infertility. It is well known that MuV could disrupt the BTB and infect major testicular cells, including SCs, Leydig cells, TMφ, and male germ cells. This can result in mumps orchitis, which is usually characterized by the congestion of the seminiferous tubules and lymphocyte infiltrations, leading to dysfunction of the testis and impairment of male infertility (Wu et al., 2016; Westphal et al., 2019). Another virus, the ZIKV, which once caused a pandemic (Epelboin et al., 2017), could be detected in the seminal fluid of infected males for extended periods of time, likely initiating orchitis and epididymitis through the induction of innate immune responses in Leydig cells and SCs, eventually leading to male infertility (Govero et al., 2016; Ma et al., 2016; Stassen et al., 2018). More importantly, SARS-CoV, which has recently caused widespread concern, was shown to be able to cause orchitis. This was determined based on the findings from six dead patients who displayed widespread germ cell destruction, few or no spermatozoon in the seminiferous tubule, and leukocyte infiltration, as well as significantly increased levels of T-cells and macrophages in the interstitial tissue (Douglas et al., 2004; Xu et al., 2006; Ma et al., 2020).

However, the main question raising a lot of concern would be whether SARS-CoV-2 can cause testicular infections similar to the viruses mentioned previously. Firstly, the observed similarities between SARS-CoV and SARS-CoV-2 found in the most immunogenic part of the virus, as described above, suggest that the human testis might be a potential target for infection with SARS-CoV-2, and it might consequently play significant roles in the function of the testis, as well as male fertility, from the viewpoint of viral biology.

A single center-based study comparing the sex hormones between 81 reproductive-aged men infected with SARS-CoV-2 and 100 age-matched healthy men showed that infection with SARS-CoV-2 could affect male gonadal function. Additionally, although the serum luteinizing hormone (LH) was significantly increased, the ratio of testosterone (T) to LH (T:LH) was dramatically decreased in males with severe COVID-19 (Ma et al., 2021). This study provided the first direct evidence regarding the influence of COVID-19 on male sex hormones, alerting more attention to the evaluation of the gonadal function among patients who recovered from infection with SARS-CoV-2, especially young men. In addition, several case-series studies have shown that SARS-CoV-2 RNA was detected in serum (Lescure et al., 2020; Young et al., 2020; Zheng et al., 2020) and urine samples (Cao, 2020; Ling et al., 2020; Wang L. et al., 2020; Zheng et al., 2020). Interestingly, SARS-CoV-2 RNA was demonstrated to be detected in the urine specimens of three patients after their throat swabs were shown to be negative (Ling et al., 2020). Again, based on the indirect evidence in plasma and urine specimens, the possibility of the impact of SARS-CoV-2 on the testis remains present and cannot be ignored. Particularly noteworthy, a clinical investigation about SARS-CoV-2 in semen demonstrated that in a total of 38 patients who provided a semen specimen, 23 participants (60.5%) had achieved clinical recovery, 15 participants (39.5%) were still at the acute stage of infection, and 6 patients (15.8%) were found to be positive for SARS-CoV-2, including 4 patients who were at the acute stage of infection and 2 patients who were recovering (Li D. et al., 2020). Another recent study found two human testis samples with severe COVID-19 to be positive for SARS-CoV-2 nucleic acid, with the testicular sections positive for SARS-CoV spike S1; and the histological morphology of the testis, in four of the five cases, showed numerous degenerated germ cells (Ma et al., 2020). These clinical results suggested that SARS-CoV-2 might directly infect the male genital tract or testes in the process of COVID-19, and it could be an underlying etiological hypothesis of associated male infertility during or following the SARS-CoV-2 outbreak. However, more reports, to date, about patients with COVID-19 who were in recovery showed that no positive SARS-CoV-2 RNA was detected in semen samples or testicular biopsy specimens (Li H. et al., 2020; Song et al., 2020). These findings suggest that positive SARS-CoV-2 RNA in semen is a rare event and is associated with severe COVID-19, even in dead patients, while it might be negative in the patients with mild or moderate COVID-19. Interestingly, Gacci et al. (2021) found that one out of five patients with SARS-CoV-2 infections and proven healing has oligo-crypto-azoospermia, despite the absence of virus RNA in semen. The majority of men with abnormal sperm parameters have biological children, excluding other factors of infertility. Indeed, treatment with drugs, including antibiotics, antiviral drugs, and immunomodulators, and fever might affect male fertility (Carlsen et al., 2003; Semet et al., 2017).

Conclusively, the issue of SARS-CoV-2 likely infecting the testis, especially the patients with severe COVID-19, cannot be ignored based on the viral, biological, and clinical data above; and the germ cell loss in the patients with COVID-19 should raise alarming attention to the potential impairment of the reproductive function of the patients, especially the reproductive-aged men.

## Clinical Remedies to Potential Male Patients With Coronavirus Disease 2019

Although the current clinical data demonstrate that male patients with COVID-19 constitute the predominantly (56–73%) infected population (Chen et al., 2020; Huang et al., 2020a; Xu et al., 2020), its potential impact on the male reproductive system is unclear. As a result, in the process of diagnosis and treatment of male patients with or without COVID-19 during a SARS-CoV-2 outbreak, not only should the common clinical manifestations of COVID-19 be timely considered according to the Guidelines issued by the WHO (World Health Organization, 2020b), but the health of the male reproductive tract should also be paid more attention to.

For male patients with COVID-19, the common clinical manifestations could be timely intervened with individualized treatments, according to the Guidelines issued by the WHO (World Health Organization, 2020b). For patients with severe symptoms, some under-investigated strategies about dampening the immune-mediated damage could be considered. For example, tocilizumab (Liu et al., 2020), a IL-6 receptor-targeted antibody, could be used to impair the impact of the inflammatory cytokine storm; mesenchymal stem cells (MSCs), exerting anti-apoptotic, anti-inflammatory, immunomodulatory, regenerative, and anti-fibrotic properties, could offer a new approach in treating the damage done in the lungs.

Beyond that, the health of the male reproductive system should also be considered. There have been few patients reported to have damaged testis, despite the data from existing studies suggesting the possibility that SARS-CoV-2 could infect Leydig cells and SCs, resulting in viral orchitis, which is not excluded completely. When male patients, especially reproductive-age male patients, would be treated in the male outpatient department due to fever, testicular pain, and other clinical symptoms during an outbreak of SARS-CoV-2, the possibility of infection with SARS-CoV-2 should be considered, and the patients should be timely screened using semen, urine, blood, and throat swabs. Besides, the physician should apply all necessary protective measures to prevent the transmission of the virus between humans. In special cases, the seminal plasma of patients could be used to detect SARS-CoV-2 RNA.

In this particular phase, we recommend that sperm donations and cryopreservation should be carried out after ruling out the risk of SARS-CoV-2 (Huang et al., 2020b; Paoli et al., 2020), as the possibility of an infection with SARS-CoV-2 exists in the male reproductive tract, and the duration of this infection cannot be specified. However, fertility services for patients with particular scenarios, including azoospermic, cryptozoospermic, and systemic autoimmune men whose “fertility window” might be transitory following medical treatment, are recommended to continue in a considered and safe manner (Esteves et al., 2020).

## Conclusion

Some previous studies and recent bioinformatics analysis showed that SARS-CoV-2 could infect ACE2-positive cells, including Leydig cells, SCs, and germ cells, impacting the function of the testis. Moreover, due to the characteristic immune function of the testis and potential immune-mediated damage caused by a cytokine storm, lymphopenia, and immunodepletion by COVID-19, male patients, especially with severe COVID-19, might exhibit impairments in fertility. Both direct and indirect existing epidemiological evidences have indicated that SARS-CoV-2 RNA could be detected in the testis, with the potential of impairing the spermatogenesis and impacting male fertility being foreseeable, which should be taken seriously. With the pandemic of COVID-19 around the world, protecting male fertility should be of heightened importance.

## Author Contributions

JX contributed to literature search, data organization, figure preparation, and manuscript writing. LH, YZ, and ZH contributed to literature search, writing, and revision. YS, YF, ZF, and MP contributed to literature search and writing. CL, HZ, and KZ contributed to all of these aspects and to conceptualization and were responsible for the entire setup and structure design of manuscript. All authors have read and agreed to the published version of the manuscript.

## Conflict of Interest

The authors declare that the research was conducted in the absence of any commercial or financial relationships that could be construed as a potential conflict of interest.
